# Age-Dependent Modifications of AMPA Receptor Subunit Expression Levels and Related Cognitive Effects in 3xTg-AD Mice

**DOI:** 10.3389/fnagi.2014.00200

**Published:** 2014-08-05

**Authors:** Pamela Cantanelli, Samantha Sperduti, Domenico Ciavardelli, Liborio Stuppia, Valentina Gatta, Stefano Luca Sensi

**Affiliations:** ^1^Molecular Neurology Unit, Center of Excellence on Aging (CeSI), “G. d’Annunzio” University, Chieti, Italy; ^2^Functional Genetics Unit, Center of Excellence on Aging (CeSI), “G. d’Annunzio” University, Chieti, Italy; ^3^Department of Psychological Sciences, School of Medicine and Health Sciences, “G. d’Annunzio” University, Chieti, Italy; ^4^School of Human and Social Science, Kore University of Enna, Enna, Italy; ^5^Department of Neuroscience and Imaging, “G. d’Annunzio” University, Chieti, Italy; ^6^Department of Neurology, Institute for Memory Impairments and Neurological Disorders, University of California Irvine, Irvine, CA, USA; ^7^Department of Pharmacology, Institute for Memory Impairments and Neurological Disorders, University of California Irvine, Irvine, CA, USA

**Keywords:** AMPA receptors, GluA1–4, gene expression, RNA editing, neurotransmission, 3xTg-AD mice, hippocampus, cognitive deficits

## Abstract

GluA1, GluA2, GluA3, and GluA4 are the constitutive subunits of amino-3-hydroxy-5-methyl-4-isoxazolepropionic acid receptors (AMPARs), the major mediators of fast excitatory transmission in the mammalian central nervous system. Most AMPARs are Ca^2+^-impermeable because of the presence of the GluA2 subunit. GluA2 mRNA undergoes an editing process that results in a Q–R substitution, a key factor in the regulation of AMPAR Ca^2+^-permeability. AMPARs lacking GluA2 or containing the unedited subunit are permeable to Ca^2+^ and Zn^2+^. The phenomenon physiologically modulates synaptic plasticity while, in pathologic conditions, leads to increased vulnerability to excitotoxic neuronal death. Given the importance of these subunits, we have therefore evaluated possible associations between changes in expression levels of AMPAR subunits and development of cognitive deficits in 3xTg-AD mice, a widely investigated transgenic mouse model of Alzheimer’s disease (AD). With quantitative real-time PCR analysis, we assayed hippocampal mRNA expression levels of GluA1–4 subunits occurring in young [3 months of age (m.o.a.)] and old (12 m.o.a) Tg-AD mice and made comparisons with levels found in age-matched wild type (WT) mice. Efficiency of GluA2 RNA editing was also analyzed. All animals were cognitively tested for learning short- and long-term spatial memory with the Morris Water Maze (MWM) navigation task. 3xTg-AD mice showed age-dependent decreases of mRNA levels for all the AMPAR subunits, with the exception of GluA2. Editing remained fully efficient with aging in 3xTg-AD and WT mice. A one-to-one correlation analysis between MWM performances and GluA1–4 mRNA expression profiles showed negative correlations between GluA2 levels and MWM performances in young 3xTg-AD mice. On the contrary, positive correlations between GluA2 mRNA and MWM performances were found in young WT mice. Our data suggest that increases of AMPARs that contain GluA1, GluA3, and GluA4 subunits may help in maintaining cognition in pre-symptomatic 3xTg-AD mice.

## Introduction

Alzheimer’s disease (AD) is a progressive neurodegenerative disorder leading to the most common form of dementia. Amyloid- and tau-dependent pathology (Goedert and Spillantini, 2006) are the main features of the disease, but impairment of glutamatergic transmission and excitotoxicity also play an important role in the pathogenic cascade that leads to synaptic dysfunction, neuronal loss, and brain atrophy (Small et al., 2001; Selkoe, 2002).

The glutamate receptor family encompasses three types of ionotropic receptors, NMDAR, amino-3-hydroxy-5-methyl-4-isoxazolepropionic acid receptors (AMPAR), and some kainate receptors as well as metabotropic receptors (mGluRs I–III) (Traynelis et al., 2010). In the mammalian central nervous system, glutamatergic receptors selectively activated by the α-AMPA mediate the majority of fast excitatory synaptic transmission (Bliss and Collingridge, 1993). AMPARs are composed of four different subunits: GluA1, GluA2, GluA3, and GluA4. AMPAR subunits are organized in homo- or heterotetrameric complexes (Borges and Dingledine, 1998). The ordered combination of AMPAR subunits leads to the formation of distinct receptors that can differ upon functional features like ion selectivity or inward rectification as well as capability to assemble in functional complexes containing scaffolding proteins and signaling molecules. AMPAR subunits, although similar in structure, show distinct variations in their C-terminal domains, a property that results due to the presence of selective sites that are targets for phosphorylation or protein–protein interactions (Shepherd and Huganir, 2007). Post-transcriptional modifications of GluA1–4 pre-mRNAs, like RNA editing or alternative splicing, further modulate AMPAR functioning.

The GluA2 subunit represents a major functional switch for the receptor as its presence confers impermeability to important divalent cations like Ca^2+^ and Zn^2+^. The GluA2 subunit undergoes a post-transcriptional editing process at the Q/R site that promotes a glutamine to arginine substitution in the channel-forming intramembrane segment. The presence of the arginine residue in the pore of the channel inhibits the permeability to divalents (Hollmann et al., 1991; Hume et al., 1991; Sommer et al., 1991; Lomeli et al., 1994; Rosenthal and Seeburg, 2012). Most AMPARs contain the GluA2 subunit and are therefore Ca^2+^-impermeable (CaI-AMPARs). However, significant expression of Ca^2+^-permeable AMPARs (CaP-AMPARs) occurs in several brain regions and in subsets (like hippocampal GABAergic interneurons or glutamatergic hippocampal pyramidal neurons) of neuronal populations, a process that is also dependent upon development (Jonas et al., 1994; Geiger et al., 1995; Ogoshi and Weiss, 2003; Cull-Candy et al., 2006). Interestingly, the AMPAR subunit composition and resulting Ca^2+^-permeability of the channels are not static processes. AMPARs undergo a constant and “on demand” dynamic remodeling that is largely driven by variations in neuronal activity or changed in response to pathological conditions (Liu and Cull-Candy, 2000, 2002; Liu and Zukin, 2007).

Amino-3-hydroxy-5-methyl-4-isoxazolepropionic acid receptors play a central role in the modulation of synaptic plasticity. A strong body of evidence has shown that regulation of AMPAR trafficking at membranes and variations in receptor functioning are critically involved in many forms of synaptic plasticity (Liu and Cull-Candy, 2002; Huganir and Nicoll, 2013). For instance, synaptic recruitment of AMPARs is required for the development of long-term potentiation (LTP), whereas a decrease in number and function of these receptors affects long-term depression (LTD) (Carroll et al., 1999; Song and Huganir, 2002; Bredt and Nicoll, 2003). The switch between CaI- and CaP-AMPARs is occurring at different stages of synaptic plasticity. CaP-AMPARs are transiently incorporated during early stages of NMDAR-dependent LTP in CA1 hippocampal pyramidal neurons and replaced with CaI-AMPARs for plasticity maintenance (Plant et al., 2006). CaP-AMPARs also play a key role in NMDAR-independent plasticity mechanisms like synaptic scaling, an homeostatic form of plasticity by which neurons restore and adjust baseline levels of activity via a slow modification in glutamatergic receptor trafficking at synaptic sites (Pérez-Otaño and Ehlers, 2005; Hou et al., 2008; Huganir and Nicoll, 2013).

Glutamate receptors are crucial for synaptic plasticity, but also possess a dark side as large influx of divalents like Ca^2+^ and Zn^2+^ through sustained activation of NMDARs and CaP-AMPARs is considered a major trigger for the initiation of the excitotoxic cascade that leads to neuronal death in a number of acute brain disorders like ischemia, brain trauma, and seizures as well as in neurodegenerative disease like amyotrophic lateral sclerosis (ALS) and AD (Lee et al., 1999; Weiss and Sensi, 2000; Weiss, 2011). In particular, overactivation of CaP-AMPARs leads to Ca^2+^ and Zn^2+^ overloads in the neuronal cytosol and in mitochondria where the two cations synergistically act to promote generation of reactive oxygen species (ROS), necrosis as well as release of pro-apoptotic mediators (Weiss and Sensi, 2000; Sensi et al., 2009). Thus, alterations in levels of GluA2 and/or CaP-AMPARs mRNA expression levels or changes in AMPAR trafficking at the post-synaptic membrane can greatly influence synaptic efficacy as well as neuronal survival (Weiss and Sensi, 2000; Cull-Candy et al., 2006; Liu and Zukin, 2007; Weiss, 2011). Deregulation in GluA2 mRNA editing at the Q/R site, a process usually extremely efficient (near 100%), has been associated with important neurotoxic consequences and shown to occur in transient global ischemia or several neurodegenerative conditions (Pellegrini-Giampietro et al., 1997; Kawahara et al., 2004; Peng et al., 2006).

Whereas the involvement of AMPARs has been widely established in several neurological disorders (Weiss, 2011), the role of these receptors in AD remains elusive. Furthermore, largely unknown is what CaP-AMPARs do in the AD brain, a major knowledge gap given the important deregulation of divalent cations that occurs in the disease (Corona et al., 2011).

To investigate this issue in a preclinical AD model, we employed a triple transgenic mouse model of AD, the 3xTg-AD. These Tg mice, by harboring three human dementia-related transgenes, APP_swe_, PS1_M146V_, and *tau*_P301L_, reproduce key AD-related features like amyloid beta- (Aβ) and tau-dependent pathology, as well as an age-dependent development of synaptic dysfunction and LTP deficits (Oddo et al., 2003). The model also shows an important acceleration of expression changes of genes that are related to aging, the other major contributing factor in the disease (Gatta et al., 2014). Both Aβ and tau pathologies occur in an age- and region-dependent manner, another feature that resembles what found in the AD brain (Mesulam, 2000) where Aβ plaques are known to precede neurofibrillary tangles (NFTs) formation (Mastrangelo and Bowers, 2008).

Thus, the aim of our study was to evaluate age-dependent changes in AMPAR gene transcriptional levels as well as GluA2 editing in a strategic region, the 3xTg-AD hippocampus.

Age-dependent modifications in GluA expression as well as GluA2 editing were studied in young (3 months of age; m.o.a.) and old (12 m.o.a) 3xTg-AD mice. Expression changes were compared to age-dependent modifications occurring in age-matched wild type (WT) mice. In the second part of the study, correlations between GluA expression and modulation of cognitive performances were also evaluated.

## Materials and Methods

### Animals

Procedures involving animals and their care were approved by the institutional Ethics Committee (CeSI protocol #: AD-301) conducted in conformity with the institutional guidelines that are in compliance with national (D.L. n. 116, G.U., suppl. 40, 18 February, 1992) and international laws and policies. All efforts were made to reduce animal number and to minimize their suffering. Triple-transgenic mice, the 3xTg-AD, overexpressing mutant APP (APPSwe), PS1 (PS1M146V), and hyper-phosphorylated tau (tauP301L), were originally obtained by Oddo et al. (2003), by coinjecting two independent human transgenes, APPSwe and tauP301L, in single-cell embryos that are harvested from mutant homozygous PS1M146V knock-in mice (PS1-KI). PS1-KI mice were originally generated on a hybrid 129/C57BL6 background (Guo et al., 1999). Hemizygous F1 mice were crossed to obtain mice homozygous for all three transgenes. These mice have been characterized by Oddo et al. (2003) and generously provided by Frank LaFerla. Thus, non-Tg mice of the same mixed 129/C57BL6 background have been used as (WT) controls.

Three months of age (m.o.a.) female 3xTg-AD and WT mice (*n* = 6 for group) together with 12 m.o.a. female 3xTg-AD and WT mice (*n* = 6 for group) were enrolled for the study. As previously indicated (Giménez-Llort et al., 2007; Sterniczuk et al., 2010), we have not observed major motor or visuomotor coordination deficits. We also did not observe major motivational problems.

### Behavioral task – Morris Water Maze

To perform the Morris Water Maze (MWM) (Morris, 1984), we employed a circular pool (1.3 m diameter) filled with water. The maze was placed in a room containing several intra and extra-maze visual landmarks. The test was performed in accordance with what described for AD mouse models (Puzzo et al., 2014). Mice were trained to swim in the pool until they climbed on a white escape platform (10 cm in diameter), located in the middle of one of the four quadrants positioned at a depth of 2 cm below water surface. Before the beginning of the first training session, mice were placed on the escape platform for 10 s in order to allow an initial orientation in the pool. If a mouse did not find the platform within 90 s, the animal was guided to it by the experimenter and allowed to remain there for an additional 10 s. Mice were given four trials per day separated by an inter-trial interval of 20 min for four consecutive days. Analysis of the learning curve in a subset of mice (3 m.o.a WT = 3*n*; 3 m.o.a. 3xTg-AD = 4*n*; 12 m.o.a. WT = 5*n*; 12 m.o.a; 12 m.o.a. 3xTg-AD = 4*n*), showed that all the animals learned the task equally well within the fourth day of learning. Spatial memory retention was evaluated 1.5 and 24 h after the end of the last training trial. Both probe trials consisted of a 60 s free swim in the pool where the platform has been removed. Parameters employed to evaluate memory skills were time to reach the platform location (latency), number of crosses over the platform location (crosses), and time spent in target (T. Target) and opposite (T. Opposite) quadrants. All the enrolled animals were tested and completed the evaluation. No differences in navigation speed were observed in old mice when comparing the two strains. Analysis of swim speed [with the SMART 2.5.21 program (Panlab, Barcelona, Spain] showed statistically non-significant differences among the two study groups as mean speed (cm/s) ± SEM was 24.99 ± 1.78 in 12.m.o.a WT mice and 27.45 ± 3.78 in age-matched 3xTg-AD mice (*p* = 0.058). Interestingly (possibly indicating a compensatory mechanism taking place in pre-symptomatic AD mice), young (3 m.o.a) 3xTg-AD mice were significantly faster with 34.00 ± 0.53 compared to age-matched WT mice (25.98 ± 1.66; *P* = 0.01).

### Quantitative real-time PCR analysis

After completion of behavioral tasks, animal were sacrificed by carbon dioxide inhalation, hippocampi dissected, kept in RNA*later* RNA Stabilization Reagent (Qiagen, Hilden, Germany), and stored at −80°C. For RNA analysis, hippocampi were homogenized by a hand glass potter and total RNA extracted using the RNeasy Microarray Tissue Mini Kit (Qiagen, Hilden, Germany) according to the manufacturer protocol. The purity and quantity of RNA were assessed by Agilent 8453 Spectrophotometer (Agilent, Santa Clara, CA, USA). One microgram of total RNA was used for cDNA synthesis performed in a 20 μl reaction through the high-capacity cDNA reverse transcription kit (Applied Biosystems, Paisley, UK). Reaction conditions were as follows: 37°C for 60 min, 95°C for 5 min, and cool at 4°C. Quantitative real-time PCR analysis (qRT-PCR) was carried out on the ABI 7900HT Sequencing Detection System (Applied Biosystems, Paisley, UK). Amplification reaction was performed in a total volume of 30 μl containing 1× TaqMan Universal PCR Master mix, no AmpErase UNG (Applied Biosystems, Paisley, UK), and 1.5 μl of target cDNA along with the Prime Time Mini qPCR Assay (Integrated DNA Technologies, Coralville, IA, USA). Mouse gene-specific primer and fluorescent probe sets were: primer Fw: 5′-CTTTGTCAAGCTCATTTCCTGG-3′, primer Rev: 5′-TCTTGCTCAGTGTCCTTGC-3′, probe 5′-CACCCTGTTGCTGTAGCCGTATTCA-3′, for glyceraldehyde 3-phosphate dehydrogenase (GAPDH); primer Fw: 5′-ACCCTCCATGTGATCGAAATG-3′, primer Rev: 5′-GGTTCTATTCTGGACGCTTGAG-3′, probe: 5′-ATAAATTTGTCCCCGCAGCCACG-3′, for GluA1; primer Fw: 5′-AAAGAATACCCTGGAGCACAC-3′, primer Rev: 5′-CCAAACAATCTCCTGCATTTCC-3′, probe: 5′-ACTTCGGCCCTGACTTATGATGCTG-3′, for GluA2; primer Fw: 5′-AGTGGGAGAAGTTTGTGTACC-3′, Rev: 5′-TGATGCGTCTGAATTCCTGG-3′, probe: 5′-ACAGAACGAGGGTTTTCCATCCTGC-3′, for GluA3; primer Fw: 5′-TTCTACATTCTGGTTGGCGG-3′, primer Rev: 5′-CCTGGCTTTGTTTCTTATGGC-3′, probe: 5′-TCAGCTTCATTCTCTTCGCCTCTGC-3′, for GluA4. Six biological replicates were performed for each study group for a total of 24 mice. All the experiments were carried out in triplicates for each data point. The positive reaction was detected by accumulation of fluorescent signals. Data analysis was performed using the *Ct value* (threshold cycle) defined as the PCR cycle at which the amplification curve intercepts the threshold fluorescence. Threshold fluorescence has been defined as the numerical value that reflects a statistically significant point above the background signal (baseline). Using the 2^−Δ^*^Ct^* method (Schmittgen and Livak, 2008), relative expression levels of GluA1–4 mRNA were calculated for each sample after normalization against GAPDH, the housekeeping gene.

### GluA2 Q/R editing

To assess GluA2 editing, we performed a sequencing analysis in agreement with the method described by Barbon et al. (2003). For each sample, PCR amplification of the GluA2 cDNA region containing the editing Q/R site was carried out using the primer pairs Fw: 5′-CGAGTGGCACACTGAGGAAT-3′ and Rev: 5′-CTCTTTAGTGGAGCCAGAGTCTAA-3′, thereby generating a fragment of 325 bp in length. All amplicons were subsequently sequenced with forward and reverse primers. A Big Dye Terminator Kit (Applied Biosystems, Paisley, UK) was employed to perform sequencing and samples then analyzed on an ABI 3130xl automatic sequencer (Applied Biosystems, Paisley, UK), an analytical tool that has an accuracy of base calling of 98.5%.

### Statistical analysis

Statistical analysis of MWM and gene expression data was performed by two-factor ANOVA followed by Fisher’s least significant difference (LSD) *post hoc* test using Statistica 6.0 software (Statsoft, Tulsa, OK, USA). Genotype and age were the independent factors. Outliers were detected by Grubbs’ test. Significance of correlations between MWM cognitive performances (latency and crosses) and qRT-PCR expression levels of AMPAR subunits were assessed by Spearman correlation analysis using MetaboAnalyst statistical analysis module (Xia and Wishart, 2011). Data were derived from each study group (i.e., 3 and 12 months old 3xTg-AD mice and 3 and 12 months old WT mice). *p* values <0.050 were considered statistically significant. Correction for multiple comparisons was performed using the false discovery rate (FDR) method. *p* values <0.050 (FDR <0.100) were considered statistically significant.

## Results

### Expression analysis of GluA1–4 by qRT-PCR

Hippocampal GluA1, GluA2, GluA3, and GluA4 transcriptional levels in 3xTg-AD mice at 3 or 12 m.o.a. as well as in age-matched WT animals were studied by qRT-PCR. Each gene level was evaluated as 2^−Δ^*^Ct^*, an index of gene expression that is normalized to the GAPDH. This reference gene showed comparable expression levels through the study groups (mean *Ct* ± 95% confidence level = 17.6 ± 0.3 and 17.7 ± 0.2 for 3 and 12 m.o.a. for WT mice, and 17.7 ± 0.2 and 17.5 ± 0.3 for 3 and 12 m.o.a. for 3xTg-AD mice; Table S1). For GluA1, expression (mean ± S.E.M.) values were 0.068 ± 0.005 in 3xTg-AD mice at 3 m.o.a. and 0.055 ± 0.009 in age-matched WT mice while in 3xTg-AD mice at 12 m.o.a values were 0.049 ± 0.007 and 0.046 ± 0.002 in age-matched WT animals. For GluA2, we found 0.030 ± 0.002 in young 3xTg-AD mice and 0.022 ± 0.005 in age-matched WT mice or 0.019 ± 0.003 in old 3xTg-AD animals and 0.037 ± 0.009 in age-matched WT animals. For GluA3, expression levels were 0.011 ± 0.001 in young 3xTg-AD mice and 0.006 ± 0.001 in age-matched WT mice or 0.006 ± 0.001 in old 3xTg-AD animals and 0.013 ± 0.002 in age-matched WT mice. For GluA4, values were 0.003 ± 0.0005 in young 3xTg-AD mice and 0.002 ± 0.0003 in age-matched WT mice or 0.002 ± 0.0001 in old 3xTg-AD animals and 0.002 ± 0.0002 in age-matched WT animals.

We then sorted effects of aging and genotype on AMPAR subunit expression levels. For clarity, results are presented as follows.

#### Effect of aging

**Figure 1:**
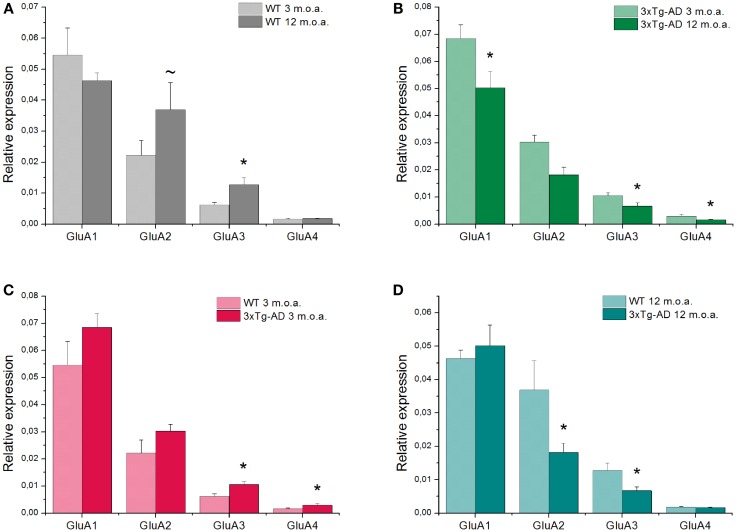
**Quantitative real-time PCR analysis of GluA1–4 genes is shown**. Graph bars depict mean expression levels of GluA1–4 relative to the internal reference. **(A)** Comparison between 12 m.o.a (old) vs. 3 m.o.a (young) animals WT mice showed an increase in GluA3 expression level (*p = 0.004). A trend toward increased levels of GluA2 expression was also found (~*p* = 0.073). **(B)** Comparison between old vs. young mice 3xTg-AD animals showed decreases in GluA1, 3, and 4 expression levels (*p = 0.045; *p = 0.047; *p = 0.011, respectively). **(C)** In AD mice, comparison between 3xTg-AD mice vs. WT mice at 3 m.o.a. showed increases in GluA3 (*p = 0.039) and GluA4 (*p = 0.030) expression levels. **(D)** Comparison between 3xTg-AD mice vs. age-matched WT mice showed a decrease in GluA2 (*p = 0.038) and GluA3 (*p = 0.005) expression levels in the 3xTg-AD animals. Data are expressed as mean values ± SEM obtained from six mice in each study group. *P* value is the result of two-factor ANOVA and Fisher’s least significant difference (LSD) *post hoc* test (*p < 0.05; ~trend 0.05 < *p* < 0.1).

GluA1–4 genes expression varied upon aging in WT and 3xTg-AD mice. In WT mice, aging promoted a significant up-regulation of GluA3 gene expression (*p* = 0.004). A similar trend, that, however, did not reach statistical significance, was observed for GluA2 (*p* = 0.073). No effects were observed for the other subunits (Figure 1A). In 3xTg-AD mice, aging promoted a statistically significant reduction of all the AMPAR subunits (GluA1, *p* = 0.045; GluA3, *p* = 0.047; GluA4, *p* = 0.011; Figure 1B) with the exception of GluA2 that remained stable (Table S2).

#### Effect of genotype

Comparison between young 3xTg-AD vs. age-matched WT mice revealed that Tg-AD animals showed a significant up-regulation of GluA3 (*p* = 0.039) and GluA4 (*p* = 0.007) whereas no changes were detected for GluA1 and GluA2 (Figure 1C).

Comparison between old 3xTg-AD mice vs. age-matched WT animals showed, in the AD mice, a significant down-regulation of GluA2 (*p* = 0.038) and GluA3 (*p* = 0.005) while expression levels of GluA1 and GluA4 were unchanged (Figure 1D; Table S2).

### Editing

The editing process of GluA2 pre-mRNAs results in the substitution of a codon for glutamine (CAG) with a codon for arginine (CGG) at the so-called Q/R site. This post-transcriptional change of GluA2 transcripts confers to AMPARs impermeability to Ca^2+^ and other divalents (Kawahara et al., 2004). Incomplete editing has been shown to occur in neuropathological conditions and we have therefore evaluated the process in our preclinical AD model.

**Figure 2:**
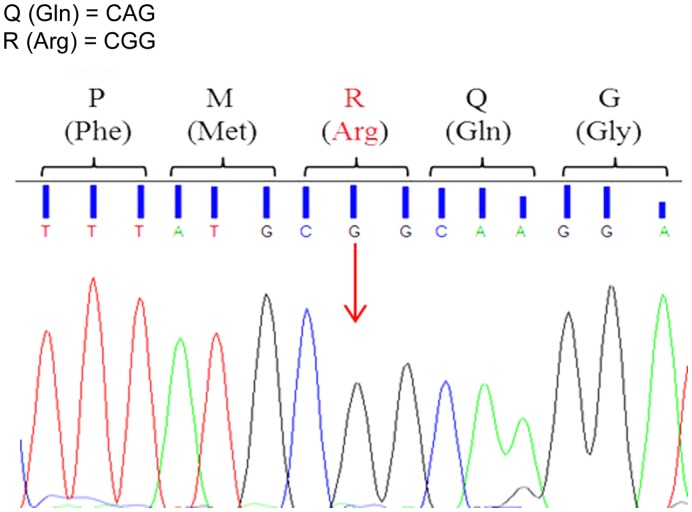
**Representative electropherogram of GluA2 Q/R editing-site sequence analysis**. All animals, 3xTg-AD and WT, showed full editing with the Adenine to Guanine (A > G) substitution in the GluA2 Q/R editing site (arrow) indicated by the presence of a black peak (G) and the absence of a green peak (A).

To assess Q/R site editing of GluA2 pre-mRNAs, each sample was sequenced using forward and reverse primers. Editing remained extremely efficient in all animals throughout development and aging. The Adenine to Guanine substitution in the Q/R site occurred in all the analyzed sequences (Figure 2). The forward and reverse sequences provided equivalent results.

### 3xTg-AD mice show cognitive deficits as assessed by MWM test

Spatial learning and memory were investigated with the MWM test, a behavioral test that evaluates hippocampal-dependent memory, one of the first cognitive functions that is lost in the course of AD (Morris, 1984; Puzzo et al., 2014). Young and old animals (at 3 and 12 m.o.a.) underwent a 4 days training period. 1.5 and 24 h after the last training trials, mice were tested with probe trials in order to evaluate short- and long-term memory performances, respectively. While all the 3xTg-AD mice eventually learned as well as the WT animals at the end of the fourth day of training, clear differences were found especially in old 3xTg-AD animals that showed impairments in the first 3 days of training (Figure S1). When compared to age-matched WT mice, old Tg-AD animals showed statistically significant differences in learning performances as expressed by latencies in the first and second day of training (Figure S1B; *p* = 0.012 and *p* = 0.032, respectively).

**Figure 3:**
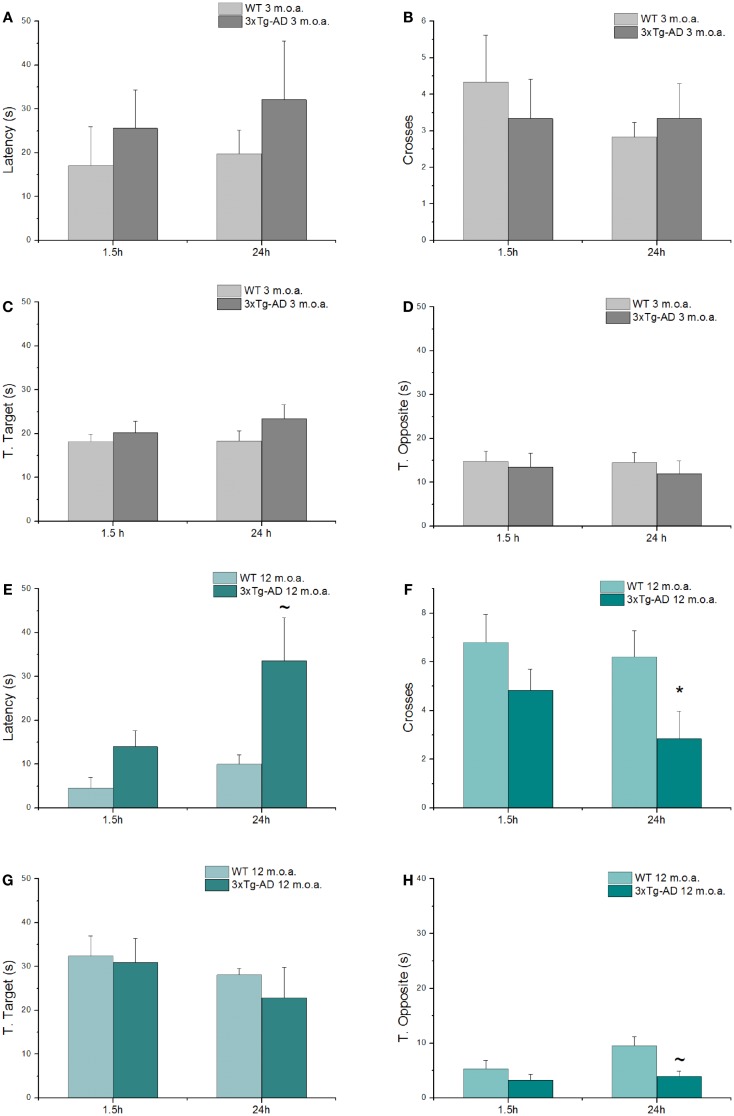
**3xTg-AD mice show cognitive deficits assessed by MWM test**. MWM performances of 3xTg-AD mice at 3 and 12 m.o.a. (*n* = 6 for group) and age-matched control mice (WT) expressed as latency **(A,E)**, number of crosses **(B,F)**, and time spent in target **(C,G)** and opposite **(D,H)** quadrants, all tested 1.5 h and at 24 h after the last training session. Compared to age-matched controls, long-term memory of old 3xTg-AD mice was impaired as indicated by statistically significant reduction in number of crosses (*p = 0.022) and increase in time (latency; ~*p* = 0.092) that animals employed to reach the point where the platform used to be. Results are expressed as mean values ± SEM. *P* value is the result of two-factor ANOVA and Fisher’s least significant difference (LSD) *post hoc* test (*p < 0.05; ~trend 0.05 < *p* < 0.1).

As expected (Oddo et al., 2003), old 3xTg-AD mice also showed deficits in long-term memory (24 h probe test; Figures 3E,F) as indicated by the statistically significant reduction in the number of crosses made over the site where the platform used to be located in the training phase (*p* = 0.023) and also exhibited a trend toward increased time spent in search of the platform (*p* = 0.092). When comparing old 3xtg-AD mice with age-matched WT animals, analysis of additional parameters showed no significant differences in T. Target scores (Figure 3G), while a trend toward significance was found in term of decreased T. Opposite scores of old 3xTg-AD mice (Figure 3H). A single item, the T. Opposite result is not indicative of a better performance as, when considered in conjunction with latencies and number of crosses, it simply indicates that old 3xTg-AD mice show deficits in reaching the platform quadrant and randomly navigate over out-of-target quadrants. No cognitive deficits were observed in young 3xTg-AD mice when compared to age-matched WT animals (Figures 3A–D; Table S3).

### Correlation analysis between mRNA GluA1–4 expression levels and MWM performances

**Figure 4:**
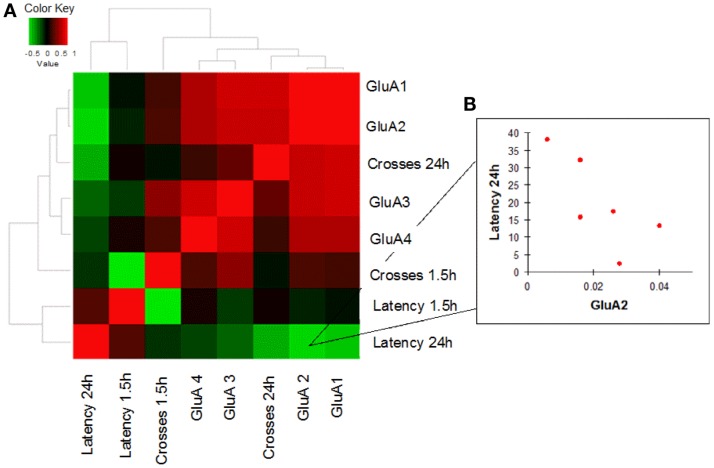
**Correlation analysis between GluA1–4 expression levels and MWM cognitive performances of WT mice is shown**. **(A)** Heatmap shows Spearman’s correlations between GluA1–4 expression levels (2^−Δ^*^Ct^*) and MWM parameters (latency and crosses) in young WT mice. Direct and inverse correlations are shown in red and green, respectively. **(B)** WT mice showed an inverse correlation between GluA2 and MWM latency at 24 h.

**Figure 5:**
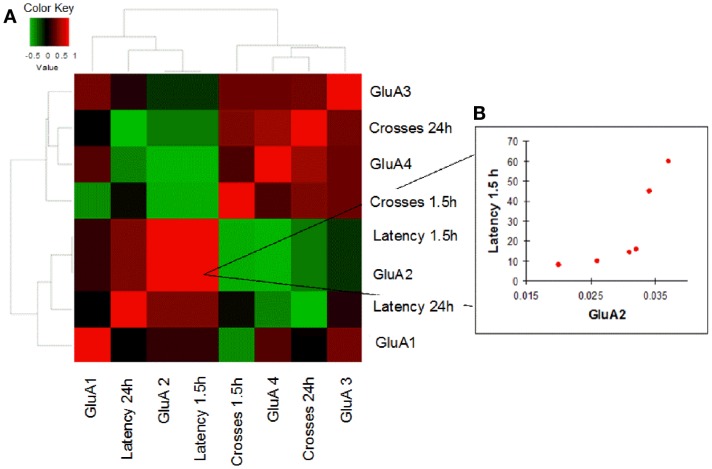
**Correlation analysis between GluA1–4 expression levels and MWM cognitive performances of 3xTg-AD mice is shown**. **(A)** Heatmap shows Spearman’s correlations between GluA1–4 expression levels (2^−Δ^*^Ct^*) and MWM parameters (latency and crosses) in young 3xTg-AD mice. Direct and inverse correlations are shown in red and green, respectively. **(B)** 3xTg-AD mice showed a direct correlation between GluA2 and MWM latency at 1.5 h.

In order to evaluate how AMPAR modulation can affect cognitive performances in the two study groups, we performed a correlation analysis between mRNA levels of each AMPAR subunit and scores (crosses and latency) of MWM parameters obtained in young and old mice. Young WT mice showed a negative correlation between GluA2 expression levels and efficient long-term memory performances as evaluated by assessing latency in the 24 h probe test (Spearman coefficient = −0.841, *p* = 0.033, FDR = 0.096; Figure 4; Table S4). Conversely, in young 3xTg-AD mice, GluA2 expression levels positively correlated with impaired short-term memory performances (as evaluated by assessing latency in the 1.5 h probe test; Spearman coefficient = 1.000, *p* = 0.003, FDR = 0.011; Figure 5; Table S5). Of note, we found that Glua2-dependent effects were found lost in aged mice, similarly to what found for the other subunits, as no significant correlations were found between MWM performances and GluA2 levels in WT and 3xTg-AD mice at 12 m.o.a (Tables S6 and S7).

## Discussion

Synaptic failure plays a critical role in the etiology of AD. A growing body of evidence indicates that Aβ oligomers, one of the most common hallmarks of AD, induce early morphofunctional and neurotransmission changes that lead to synaptic depression (Lambert et al., 1998; Hsia et al., 1999; Klein et al., 2001; Hardy and Selkoe, 2002; Klein, 2002; Kamenetz et al., 2003; Walsh and Selkoe, 2004). Aβ oligomers have been indicated to act on AMPARs to affect LTP (Parameshwaran et al., 2007; Yamin, 2009). Previous studies have shown that direct application of Aβ oligomers on hippocampal slices promote a reduction in amplitude and frequency of miniature excitatory post-synaptic current (mEPSC) in addition to trigger decreases in AMPAR open probability (Parameshwaran et al., 2007; Yamin, 2009). Oligomeric Aβ also promotes, caspase 3-dependent, removal of synaptic AMPARs, a process that, by itself, can induce spine loss and enhances LTD (Hsieh et al., 2006; Li et al., 2009; D’Amelio et al., 2011; Hu et al., 2012).

With this rationale, we have investigated whether changes of AMPAR subunit transcriptional levels may occur in 3xTg-AD mice, a preclinical model that reproduces the main pathological features of the AD brain.

In the hippocampus, a strategic and AD-targeted region, 3xTg-AD mice showed more prominent expression of GluA1 and GluA2 mRNAs while expression of GluA3 was lower and minimal levels of GluA4 were found. The GluA subunit presence is in agreement with prior studies that described a largely bimodal distribution of AMPARs, a process that is physiologically occurring in the hippocampus of non-transgenic mice (Craig et al., 1993; Wenthold et al., 1996; Black, 2005; Bassani et al., 2013).

According to that systematization, the first AMPAR population encompasses channels made of GluA1–GluA2 subunits while the second population is represented by receptors composed of GluA2–GluA3 subunits. Largely less represented are GluA1–GluA3 AMPARs or homomeric GluA1 receptors.

Compared to age-matched WT mice, old 3xTg-AD mice showed a GluA2 down-regulation (Figure 1D). Assessment of age-dependent changes in transcriptional levels for GluA1–4 indicated that, through development, the two strains may have implemented different strategies to maintain a prevalence of CaI-AMPARs.

In WT mice, we observed an aging-dependent increase in GluA2 expression whereas, in 3xTg-AD animals, aging promoted down-regulation of all the GluA subunits with the exception of GluA2, the only subunit that remained unchanged (Figures 1A,B).

In the 3xTg-AD mice, the emerging picture is that 3xTg-AD mice exhibited an overall decrease of AMPARs (Figure 1B), but at least preserved Ca^2+^-impermeability of their AMPARs. The phenomenon can be considered as a potential defensive strategy against CaP-AMPAR-driven excitotoxicity, but leaves open the possibility of defective AMPAR-dependent modulation of hippocampal synchronization.

The reduced number of AMPARs in old 3xTg-AD mice can have important consequences and be coupled with plasticity deficits as reduction in the number of these receptors can impair the desilencing of silent synapses (Hanse et al., 2013), a crucial mechanism that regulates LTP and LTD (Isaac et al., 1995). According to the model (Isaac et al., 1995), silent synapses show relative abundance of NMDARs but lack a significant amount of AMPARs. In this context, AMPARs mediate the depolarization that is critically needed to remove the voltage-dependent Mg^2+^ blockade of NMDARs, a key step required to achieve efficient glutamatergic neurotransmission (Isaac et al., 1995). The age-related modification in GluA mRNA levels that we have observed matched the cognitive deficits found in our old 3xTg-AD mice (Figures 3C,D).

Amino-3-hydroxy-5-methyl-4-isoxazolepropionic acid receptor alterations also occur early in 3xTg-AD mice. Indeed, in 3xTg-AD mice at 3 m.o.a., we found up-regulation of GluA3 and GluA4 expression while GluA1 and GluA2 remained stable (Figure 1C), thereby suggesting a functional switch toward different heterotetrameric AMPAR configurations.

Changes in GluA3 levels are intriguing. The subunit is increased in young AD mice but then decreases with aging in 3xTg-AD mice at 12 m.o.a. (Figure 1B). WT animals showed the opposite phenomenon with the development of age-dependent GluA3 increases (Figure 1A). A functional interpretation of the phenomenon should take into account that increased levels of GluA3 [along with transmembrane AMPAR regulatory proteins (TARPs)] lead to potentiation of synaptic transmission and LTD facilitation (Blair et al., 2013). Thus, GluA3 rises observed in young 3xTg-AD mice could represent an attempt to compensate defective glutamatergic transmission.

As for GluA4, although the subunit expression appears to be minimal, levels were found nevertheless increased in young 3xTg-AD mice. The phenomenon, again, could be read as compensatory as the subunit is critical to reduce AMPAR desensitization time (Geiger et al., 1995; Black, 2005), and therefore, increased GluA4 levels may lead to enhanced glutamatergic transmission. No changes in young 3xTg-AD mice appeared to occur when considering the ratio between CaI- and CaP-AMPARs as expression levels of GluA2 were found to be not significantly changed.

Correlation analysis between GluA2 transcription levels and cognitive performances as assessed with MWM tests highlighted intra-individuals differences that occurred with opposite outputs in young mice of the two strains, thereby leading to intriguing inferences on the functional role played by the subunit.

In young WT mice, high levels of GluA2 expression were associated with better long-term memory performances (Figure 4). One can speculate that this cognitive improvement is related to the known capability of GluA2 to increase spine size and density as well as to promote higher receptor stability at post-synaptic sites and induce LTP (Passafaro et al., 2003; Plant et al., 2006; Ripley et al., 2011; Hanse et al., 2013).

Interestingly, compared to WT animals, AMPAR/behavior correlations occurring in young 3xTg-AD mice were found to go in the opposite direction. The Tg-AD mice that showed higher levels of GluA2 (and therefore increased number of CaI AMPARs and lower of CaP AMPARs) were also the one exhibiting more impaired short-term memory performances (Figure 5B). The possible interpretation of the phenomenon is complex and should take into account expression levels of CaP AMPARs in two critical hippocampal subpopulations: the glutamatergic pyramidal neurons and the GABAergic interneurons.

In this context, one can speculate that, young 3xTg-AD mice with lower levels of CaP AMPAR have less efficient memory performances because of the lost positive effects of the receptors in the modulation of AMPAR-dependent synaptic plasticity. This is a crucial issue when the loss is occurring in pyramidal neurons and especially in the context of AD-related cognitive deficits.

High number of these highly Ca^2+^-permeable AMPARs can in fact compensate for Aβ-related functional impairment of NMDARs, the other major route for glutamate-driven Ca^2+^ entry (Muller et al., 1988; Watt et al., 2004). Indeed, several studies have indicated defective NMDAR functioning as a critical factor in triggering synaptic depression driven by Aβ (Kamenetz et al., 2003; Snyder et al., 2005; Yamin, 2009). The phenomenon may be related to Aβ binding to NMDARs, a mechanism that promotes altered Ca^2+^ homeostasis (De Felice et al., 2007; Shankar et al., 2007) or by the selective binding of Aβ oligomers to the NMDAR subunit, GluN2B. In that respect, Aβ binding to GluN2B can favor conformational changes that impair NMDAR-dependent signal transduction (Kessels et al., 2013). Thus, these findings support the hypothesis that 3xTg-AD mice may have defective NMDAR-dependent glutamatergic transmission, an occurrence leading to decreased Ca^2+^-dependent signaling and possibly compensated by sustained influx of the cation through GluA2-lacking AMPARs.

On the other hand, lack of CaP AMPARs may lead to decreased functioning of GABAergic interneurons, an inhibitory population that is known to possess high levels of these receptors. In that context, an AMPAR-dependent decreased activity of GABAergic interneurons can result in an out-of-synch hippocampus that further promotes network instability and cognitive dysfunction as well as a potentially excitotoxic overdrive.

This is particularly relevant when considering that, in preclinical models of AD, including the 3xTg-AD mice, hippocampal hyperactivation occurs before the appearance of Aβ plaque deposition (Busche et al., 2012; Davis et al., 2014), a phenomenon also described in AD patients (Bakker et al., 2012; Vossel et al., 2013).

In short, in 3xTg-AD mice that were poorly performing, a reduction in CaP AMPAR expression in GABAergic neurons may have brought to less efficient synchronization of hippocampal networks and led to cognitive deficits. On the same token, a decrease in the number of these receptors in pyramidal neurons could have at least promoted neuroprotection by reducing a major route for excitotoxicity.

Interestingly, the GluA-2/behavioral correlations observed in the two strains in young mice appear to be lost with aging of the animals (Tables S6 and S7). A possible speculation relies on the fact that aging induces a plethora of effects that could diminish and/or overpower the weight of GluA1–4 changes in the modulation of memory performances.

In our study, we did not explore potential effects of the MWM training and tests in the modulation of GluA1–4 mRNA levels. We have not evaluated untrained Tg-AD or control mice, and therefore, we cannot exclude that some of the GluA changes were in part dependent on MWM training. The issue warrants further studies.

Our results do not support the hypothesis that, like in other neurological conditions, the pro-AD background of our Tg-AD mice can trigger an age-dependent increase in the number of CaP-AMPARs. In 3xTg-AD mice, we did not found age-dependent changes in GluA2 levels or in the subunit editing. This finding is not in line with a recent study that showed a slight reduction in editing efficiency in the hippocampus of AD patients (Gaisler-Salomon et al., 2014). The discrepancy is likely explained by the obvious differences between preclinical AD models and the AD brain.

Of note, an additional point that is emerging is that, although 3xTg-AD mice eventually did learn the MWM task as control animals, the learning curve was slower and they showed impairments in intermediate time points of the training. This phenomenon suggests that our AD mice may have implemented different strategies to successfully accomplish the task or, perhaps, exhibited different responses to neuroendocrine modulators that are known to affect AMPAR trafficking as well as plasticity in behavioral tests like the MWM (Oitzl and de Kloet, 1992; Conboy and Sandi, 2010). For instance, behavioral tests set in motion stress- and glucocorticoid-dependent responses that induce a facilitation of learning and memory formation through increased GluA2 trafficking (Sandi, 1998; Conboy and Sandi, 2010).

In addition, it is intriguing to speculate that the learning modality exhibited by the 3xTg-AD mice may also represent an indirect index of the status of the animal cognitive reserve. The fact that old 3xTg-AD mice eventually showed no learning deficits at the fourth day of training leads to infer a more exploited use of this resource in the animals.

In summary, present findings lend support to the idea that, upon disease progression, critical modifications of glutamatergic transmission may occur in the AD brain. In that context, and always keeping in mind the limitations of preclinical AD models, our study identifies a distinct AMPAR-mediated component.

In the framework of AD-related glutamatergic deficits, further investigation is warrant to identify better and more selective pharmacological tools that can help in modulating the delicate ratio between AMPAR- and NMDAR-dependent transmission. For instance, in this regard, it could be interesting to explore the efficacy of ampakines as these AMPAR modulators can enhance synaptic plasticity and improve cognitive performances (Stäubli et al., 1994; Ingvar et al., 1997).

Furthermore, at this point, it is not possible to pin down whether AMPAR subunit expression was able to modify behavioral response by acting on many or just one of the critical hippocampal subregions (CA1, CA3, and dentate gyrus). However, in this first attempt to correlate hippocampal GluA levels with behavior, we decided to have a more robust output by using the whole hippocampus. Future studies are needed to explore correlations between behavior and AMPAR distribution in key hippocampal subregions.

## Conflict of Interest Statement

The authors declare that the research was conducted in the absence of any commercial or financial relationships that could be construed as a potential conflict of interest.

## Supplementary Material

The Supplementary Material for this article can be found online at http://www.frontiersin.org/Journal/10.3389/fnagi.2014.00200/abstract

## References

[B1] Bakker A. Gregory L. Krauss M. Albert S. Caroline L. Speck L. (2012). Reduction of hippocampal hyperactivity improves cognition in amnestic mild cognitive impairment. Neuron 74, 467–474 10.1016/j.neuron.2012.03.023 22578498 PMC3351697

[B2] Barbon A. Vallini I. La Via L. Marchina E. Barlati S. (2003). Glutamate receptor RNA editing: a molecular analysis of GluR2, GluR5 and GluR6 in human brain tissues and in NT2 cells following in vitro neural differentiation. Mol. Brain Res. 117, 168–178 10.1016/S0169-328X(03)00317-6 14559151

[B3] Bassani S. Alessandra F. Jonathan Z. Maria P. (2013). AMPAR trafficking in synapse maturation and plasticity. Cell. Mol. Life Sci. 70, 4411–4430 10.1007/s00018-013-1309-1 23475111 PMC11113961

[B4] Black M. D. (2005). Therapeutic potential of positive AMPA modulators and their relationship to AMPA receptor subunits. A review of preclinical data. Psychopharmacology 179, 154–163 10.1007/s00213-004-2065-6 15672275

[B5] Blair M. G. Nhu N.-Q. Nguyen S. H. Albani M. L’Etoile M. Andrawis M. M. (2013). Developmental changes in structural and functional properties of hippocampal AMPARs parallels the emergence of deliberative spatial navigation in juvenile rats. J. Neurosci. 33, 12218–12228 10.1523/JNEUROSCI.4827-12.2013 23884930 PMC4471169

[B6] Bliss T. V. Collingridge G. L. (1993). A synaptic model of memory: long-term potentiation in the hippocampus. Nature 361, 31–39 10.1038/361031a0 8421494

[B7] Borges K. Dingledine R. (1998). AMPA receptors: molecular and functional diversity. Prog. Brain Res. 116, 153–170 10.1016/S0079-6123(08)60436-7 9932376

[B8] Bredt D. S. Nicoll R. A. (2003). AMPA receptor trafficking at excitatory synapses. Neuron 40, 361–379 10.1016/S0896-6273(03)00640-8 14556714

[B9] Busche M. A. Chen X. Henning H. A. Reichwald J. Staufenbiel M. Sakmann B. (2012). Critical role of soluble amyloid-β for early hippocampal hyperactivity in a mouse model of Alzheimer’s disease. Proc. Natl. Acad. Sci. U.S.A. 109, 8740–8745 10.1073/pnas.1206171109 22592800 PMC3365221

[B10] Carroll R. C. Lissin D. V. von Zastrow M. Nicoll R. A. Malenka R. C. (1999). Rapid redistribution of glutamate receptors contributes to long-term depression in hippocampal cultures. Nat. Neurosci. 2, 454–460 10.1038/8123 10321250

[B11] Conboy L. Sandi C. (2010). Stress at learning facilitates memory formation by regulating AMPA receptor trafficking through a glucocorticoid action. Neuropsychopharmacology 35, 674–685 10.1038/npp.2009.172 19890264 PMC3055605

[B12] Corona C. Pensalfini A. Frazzini V. Sensi S. L. (2011). New therapeutic targets in Alzheimer’s disease: brain deregulation of calcium and zinc. Cell Death Dis. 2, e176 10.1038/cddis.2011.57 21697951 PMC3168999

[B13] Craig A. M. Blackstone C. D. Huganir R. L. Banker G. (1993). The distribution of glutamate receptors in cultured rat hippocampal neurons: postsynaptic clustering of AMPA-selective subunits. Neuron 10, 1055–1068 10.1016/0896-6273(93)90054-U 7686378

[B14] Cull-Candy S. Kelly L. Farrant M. (2006). Regulation of Ca2+-permeable AMPA receptors: synaptic plasticity and beyond. Curr. Opin. Neurobiol. 16, 288–297 10.1016/j.conb.2006.05.012 16713244

[B15] D’Amelio M. Cavallucci V. Middei S. Marchetti C. Pacioni S. Ferri A. (2011). Caspase-3 triggers early synaptic dysfunction in a mouse model of Alzheimer’s disease. Nat. Neurosci. 14, 69–76 10.1038/nn.2709 21151119

[B16] Davis K. E. Fox S. Gigg J. (2014). Increased hippocampal excitability in the 3xTgAD mouse model for Alzheimer’s disease in vivo. PLoS ONE 9:e91203 10.1371/journal.pone.0091203 24621690 PMC3951322

[B17] De Felice F. G. Velasco P. T. Lambert M. P. Viola K. Fernandez S. J. Ferreira S. T. (2007). Abeta oligomers induce neuronal oxidative stress through an N-methyl-d-aspartate receptor-dependent mechanism that is blocked by the Alzheimer drug memantine. J. Biol. Chem. 282, 11590–11601 10.1074/jbc.M607483200 17308309

[B18] Gaisler-Salomon I. Kravitz E. Feiler Y. Safran M. Biegon A. Amariglio N. (2014). Hippocampus-specific deficiency in RNA editing of GluA2 in Alzheimer’s disease. Neurobiol. Aging 35, 1785–1791 10.1016/j.neurobiolaging.2014.02.018 24679603

[B19] Gatta V. D’Aurora M. Granzotto A. Stuppia L. Sensi S. L. (2014). Early and sustained altered expression of aging-related genes in young 3xTg-AD mice. Cell Death Dis. 5, e1054 10.1038/cddis.2014.11 24525730 PMC3944230

[B20] Geiger J. R. P. Melcher T. Koh D. S. Sakmann B. Seeburg P. H. Jonas P. (1995). Relative abundance of subunit mRNAs determines gating Ca2+ permeability of AMPA receptors in principal neurons and interneurons in rat CNS. Neuron 15, 193–204 10.1016/0896-6273(95)90076-4 7619522

[B21] Giménez-Llort L. Blázquez G. Cañete T. Johansson B. Oddo S. Tobeña A. (2007). Modeling behavioral and neuronal symptoms of Alzheimer’s disease in mice: a role for intraneuronal amyloid. Neurosci. Biobehav. Rev. 31, 125–147 10.1016/j.neubiorev.2006.07.007 17055579

[B22] Goedert M. Spillantini M. G. (2006). A century of Alzheimer’s disease. Science 314, 777–781 10.1126/science.1132814 17082447

[B23] Guo Q. Fu W. Sopher B. L. Miller M. W. Ware C. B. Martin G. M. (1999). Increased vulnerability of hippocampal neurons to excitotoxic necrosis in presenilin-1 mutant knock-in mice. Nat. Med. 5, 101–106 10.1038/4789 9883847

[B24] Hanse E. Seth H. Riebe I. (2013). AMPA-silent synapses in brain development and pathology. Nat. Rev. Neurosci. 14, 839–850 10.1038/nrn3642 24201185

[B25] Hardy J. Selkoe D. J. (2002). The amyloid hypothesis of Alzheimer’s disease: progress and problems on the road to therapeutics. Science 297, 353–356 10.1126/science.1072994 12130773

[B26] Hollmann M. Hartley M. Heinemann S. (1991). Ca2+ permeability of KA-AMPA – gated glutamate receptor channels depends on subunit composition. Science 252, 851–853 10.1126/science.1709304 1709304

[B27] Hou Q. Zhang D. Jarzylo L. Huganir R. L. Man H.-Y. (2008). Homeostatic regulation of AMPA receptor expression at single hippocampal synapses. Proc. Natl. Acad. Sci. U.S.A. 105, 775–780 10.1073/pnas.0706447105 18174334 PMC2206612

[B28] Hsia A. Y. Masliah E. McConlogue L. Yu G. Q. Tatsuno G. Hu K. (1999). Plaque-independent disruption of neural circuits in Alzheimer’s disease mouse models. Proc. Natl. Acad. Sci. U.S.A. 96, 3228–3233 10.1073/pnas.96.6.3228 10077666 PMC15924

[B29] Hsieh H. Boehm J. Sato C. Iwatsubo T. Tomita T. Sisodia S. (2006). AMPAR removal underlies Abeta-induced synaptic depression and dendritic spine loss. Neuron 52, 831–843 10.1016/j.neuron.2006.10.035 17145504 PMC1850952

[B30] Hu N.-W. Ondrejcak T. Rowan M. J. (2012). Glutamate receptors in preclinical research on Alzheimer’s disease: update on recent advances. Pharmacol. Biochem. Behav. 100, 855–862 10.1016/j.pbb.2011.04.013 21536064

[B31] Huganir R. L. Nicoll R. A. (2013). AMPARs and synaptic plasticity: the last 25 years. Neuron 80, 704–717 10.1016/j.neuron.2013.10.025 24183021 PMC4195488

[B32] Hume R. I. Dingledine R. Heinemann S. F. (1991). Identification of a site in glutamate receptor subunits that controls calcium permeability. Science 253, 1028–1031 10.1126/science.1653450 1653450

[B33] Ingvar M. Ambros-Ingerson J. Davis M. Granger R. Kessler M. Rogers G. A. (1997). Enhancement by an ampakine of memory encoding in humans. Exp. Neurol. 146, 553–559 10.1006/exnr.1997.6581 9270067

[B34] Isaac J. T. Nicoll R. A. Malenka R. C. (1995). Evidence for silent synapses: implications for the expression of LTP. Neuron 15, 427–434 10.1016/0896-6273(95)90046-2 7646894

[B35] Jonas P. Racca C. Sakmann B. Seeburg P. H. Monyer H. (1994). Differences in Ca2+ permeability of AMPA-type glutamate receptor channels in neocortical neurons caused by differential GluR-B subunit expression. Neuron 12, 1281–1289 10.1016/0896-6273(94)90444-8 8011338

[B36] Kamenetz F. Tomita T. Hsieh H. Seabrook G. Borchelt D. Iwatsubo T. (2003). APP processing and synaptic function. Neuron 37, 925–937 10.1016/S0896-6273(03)00124-7 12670422

[B37] Kawahara Y. Ito K. Sun H. Aizawa H. Kanazawa I. Kwak S. (2004). RNA editing and death of motor neurons. Nature 427, 801 10.1038/427801a 14985749

[B38] Kessels H. W. Nabavi S. Malinow R. (2013). Metabotropic NMDA receptor function is required for β-amyloid-induced synaptic depression. Proc. Natl. Acad. Sci. U.S.A. 110, 4033–4038 10.1073/pnas.1219605110 23431156 PMC3593880

[B39] Klein W. L. (2002). ADDLs & protofibrils – the missing links? Neurobiol. Aging 23, 231–235 10.1016/S0197-4580(01)00312-8 11804707

[B40] Klein W. L. Krafft G. A. Finch C. E. (2001). Targeting small A β oligomers: the solution to an Alzheimer’s disease conundrum? Trends Neurosci. 24, 219–224 10.1016/S0166-2236(00)01749-5 11250006

[B41] Lambert M. P. Barlow A. K. Chromy B. A. Edwards C. Freed R. Liosatos M. (1998). Diffusible, nonfibrillar ligands derived from Abeta1-42 are potent central nervous system neurotoxins. Proc. Natl. Acad. Sci. U.S.A. 95, 6448–6453 10.1073/pnas.95.11.6448 9600986 PMC27787

[B42] Lee J. M. Zipfel G. J. Choi D. W. (1999). The changing landscape of ischaemic brain injury mechanisms. Nature 399, A7–A14 10.1038/19819 10392575

[B43] Li S. Hong S. Shepardson N. E. Walsh D. M. Shankar G. M. Selkoe D. (2009). Soluble oligomers of amyloid beta protein facilitate hippocampal long-term depression by disrupting neuronal glutamate uptake. Neuron 62, 788–801 10.1016/j.neuron.2009.05.012 19555648 PMC2702854

[B44] Liu S. J. Cull-Candy S. G. (2002). Activity-dependent change in AMPA receptor properties in cerebellar stellate cells. J. Neurosci. 22, 3881–3889 12019307 10.1523/JNEUROSCI.22-10-03881.2002 PMC6757644

[B45] Liu S. J. Zukin R. S. (2007). Ca2+-permeable AMPA receptors in synaptic plasticity and neuronal death. Trends Neurosci. 30, 126–134 10.1016/j.tins.2007.01.006 17275103

[B46] Liu S. Q. Cull-Candy S. G. (2000). synaptic activity at calcium-permeable AMPA receptors induces a switch in receptor subtype. Nature 405, 454–458 10.1038/35016590 10839540

[B47] Lomeli H. Mosbacher J. Melcher T. Höger T. Geiger J. R. Kuner T. (1994). Control of kinetic properties of AMPA receptor channels by nuclear RNA editing. Science 266, 1709–1713 10.1126/science.7992055 7992055

[B48] Mastrangelo M. A. Bowers W. J. (2008). Detailed immunohistochemical characterization of temporal and spatial progression of Alzheimer’s disease-related pathologies in male triple-transgenic mice. BMC Neurosci. 9:81 10.1186/1471-2202-9-81 18700006 PMC2527610

[B49] Mesulam M. M. (2000). A plasticity-based theory of the pathogenesis of Alzheimer’s disease. Ann. N. Y. Acad. Sci. 924, 42–52 10.1111/j.1749-6632.2000.tb05559.x 11193801

[B50] Morris R. (1984). Developments of a water-maze procedure for studying spatial learning in the rat. J. Neurosci. Methods 11, 47–60 10.1016/0165-0270(84)90007-4 6471907

[B51] Muller D. Joly M. Lynch G. (1988). Contributions of quisqualate and NMDA receptors to the induction and expression of LTP. Science 242, 1694–1697 10.1126/science.2904701 2904701

[B52] Oddo S. Caccamo A. Shepherd J. D. Murphy M. P. Golde T. E. Kayed R. (2003). Triple-transgenic model of Alzheimer’s disease with plaques and tangles: intracellular Ab and synaptic dysfunction. Neuron 39, 409–421 10.1016/S0896-6273(03)00434-3 12895417

[B53] Ogoshi F. Weiss J. H. (2003). Heterogeneity of Ca2+-permeable AMPA/kainate channel expression in hippocampal pyramidal neurons: fluorescence imaging and immunocytochemical assessment. J. Neurosci. 23, 10521–10530 14627636 10.1523/JNEUROSCI.23-33-10521.2003 PMC6740912

[B54] Oitzl M. S. de Kloet E. R. (1992). Selective corticosteroid antagonists modulate specific aspects of spatial orientation learning. Behav. Neurosci. 106, 62–71 10.1037/0735-7044.106.1.62 1313244

[B55] Parameshwaran K. Sims C. Kanju P. Vaithianathan T. Shonesy B. C. Dhanasekaran M. (2007). Amyloid B-peptide Ab 1–42 but not Ab 1–40 attenuates synaptic AMPA receptor function. Synapse 374, 367–374 10.1002/syn.20386 17372971

[B56] Passafaro M. Nakagawa T. Sala C. Sheng M. (2003). Induction of dendritic spines by an extracellular domain of AMPA receptor subunit GluR2. Nature 424, 677–681 10.1038/nature01781 12904794

[B57] Pellegrini-Giampietro D. E. Gorter J. A. Bennett M. V. Zukin R. S. (1997). The GluR2 (GluR-B) hypothesis: Ca(2+)-permeable AMPA receptors in neurological disorders. Trends Neurosci. 20, 464–470 10.1016/S0166-2236(97)01100-4 9347614

[B58] Peng P. L. Zhong X. Tu W. Soundarapandian M. M. Molner P. Zhu D. (2006). ADAR2-Dependent RNA editing of AMPA receptor subunit GluR2 determines vulnerability of neurons in forebrain ischemia. Neuron 49, 719–733 10.1016/j.neuron.2006.01.025 16504947

[B59] Pérez-Otaño I. Ehlers M. D. (2005). Homeostatic plasticity and NMDA receptor trafficking. Trends Neurosci. 28, 229–238 10.1016/j.tins.2005.03.004 15866197

[B60] Plant K. Pelkey K. A. Bortolotto Z. A. Morita D. Terashima A. McBain C. J. (2006). Transient incorporation of native GluR2-lacking AMPA receptors during hippocampal long-term potentiation. Nat. Neurosci. 9, 602–604 10.1038/nn1678 16582904

[B61] Puzzo D. Lee L. Palmeri A. Calabrese G. Arancio O. (2014). Behavioral assays with mouse models of Alzheimer’s disease: practical considerations and guidelines. Biochem. Pharmacol. 88, 450–467 10.1016/j.bcp.2014.01.011 24462904 PMC4014001

[B62] Ripley B. Otto S. Tiglio K. Williams M. E. Ghosh A. (2011). Regulation of synaptic stability by AMPA receptor reverse signaling. Proc. Natl. Acad. Sci. U.S.A. 108, 367–372 10.1073/pnas.1015163108 21173224 PMC3017209

[B63] Rosenthal J. J. Seeburg P. H. (2012). A-to-I RNA editing: effects on proteins key to neural excitability. Neuron 74, 432–439 10.1016/j.neuron.2012.04.010 22578495 PMC3724421

[B64] Sandi C. (1998). The role and mechanisms of action of glucocorticoid involvement in memory storage. Neural Plast. 6, 41–52 10.1155/NP.1998.41 9920681 PMC2565310

[B65] Schmittgen T. D. Livak K. J. (2008). Analyzing real-time PCR data by the comparative C(T) method. Nat. Protoc. 3, 1101–1108 10.1038/nprot.2008.73 18546601

[B66] Selkoe D. J. (2002). Alzheimer’s disease is a synaptic failure. Science 298, 789–791 10.1126/science.1074069 12399581

[B67] Sensi S. L. Paoletti P. Bush A. I. Sekler I. (2009). Zinc in the physiology and pathology of the CNS. Nat. Rev. Neurosci. 10, 780–791 10.1038/nrn2734 19826435

[B68] Shankar G. M. Bloodgood B. L. Townsend M. Walsh D. M. Selkoe D. J. Sabatini B. L. (2007). Natural oligomers of the Alzheimer amyloid-beta protein induce reversible synapse loss by modulating an NMDA-type glutamate receptor-dependent signaling pathway. J. Neurosci. 27, 2866–2875 10.1523/JNEUROSCI.4970-06.2007 17360908 PMC6672572

[B69] Shepherd J. D. Huganir R. L. (2007). The cell biology of synaptic plasticity: AMPA receptor trafficking. Annu. Rev. Cell Dev. Biol. 23, 613–643 10.1146/annurev.cellbio.23.090506.123516 17506699

[B70] Small D. H. Mok S. S. Bornstein J. C. (2001). Alzheimer’s disease and Abeta toxicity: from top to bottom. Nat. Rev. Neurosci. 2, 595–598 10.1038/35086072 11484003

[B71] Snyder E. M. Nong Y. Almeida C. G. Paul S. Moran T. Choi E. Y. (2005). Regulation of NMDA receptor trafficking by amyloid-beta. Nat. Neurosci. 8, 1051–1058 10.1038/nn1503 16025111

[B72] Sommer B. Kohler M. Sprengel R. Seeburg P. H. (1991). RNA editing in brain controls a determinant of ion flow in glutamate-gated channels. Cell 67, 11–19 10.1016/0092-8674(91)90568-J 1717158

[B73] Song I. Huganir R. L. (2002). Regulation of AMPA receptors during synaptic plasticity. Trends Neurosci. 25, 578–588 10.1016/S0166-2236(02)02270-1 12392933

[B74] Stäubli U. Perez Y. Xu F. B. Rogers G. Ingvar M. Stone-Elander S. (1994). Centrally active modulators of glutamate receptors facilitate the induction of long-term potentiation in vivo. Proc. Natl. Acad. Sci. U.S.A. 91, 11158–11162 10.1073/pnas.91.23.11158 7972026 PMC45186

[B75] Sterniczuk R. Antle M. C. Laferla F. M. Dyck R. H. (2010). Characterization of the 3xTg-AD mouse model of Alzheimer’s disease: part 2. Behavioral and cognitive changes. Brain Res. 1348, 149–155 10.1016/j.brainres.2010.06.011 20558146

[B76] Traynelis S. F. Wollmuth L. P. McBain C. J. Menniti F. S. Vance K. M. Ogden K. K. (2010). Glutamate receptor ion channels: structure, regulation, and function. Pharmacol. Rev. 62, 405–496 10.1124/pr.109.002451 20716669 PMC2964903

[B77] Vossel K. A. Beagle A. J. Rabinovici G. D. Shu H. Lee S. E. Naasan G. (2013). Seizures and epileptiform activity in the early stages of Alzheimer disease. JAMA Neurol. 70, 1158–1166 10.1001/jamaneurol.2013.136 23835471 PMC4013391

[B78] Walsh D. M. Selkoe D. J. (2004). Deciphering the molecular basis of memory failure in Alzheimer’s disease. Neuron 44, 181–193 10.1016/j.neuron.2004.09.010 15450169

[B79] Watt A. J. Sjöström P. J. Häusser M. Nelson S. B. Turrigiano G. G. (2004). A proportional but slower NMDA potentiation follows AMPA potentiation in LTP. Nat. Neurosci. 7, 518–524 10.1038/nn1220 15048122

[B80] Weiss J. H. (2011). Ca permeable AMPA channels in diseases of the nervous system. Front. Mol. Neurosci. 4:42 10.3389/fnmol.2011.00042 22102834 PMC3214733

[B81] Weiss J. H. Sensi S. L. (2000). Ca2+-Zn2+ permeable AMPA or kainate receptors: possible key factors in selective neurodegeneration. Trends Neurosci. 23, 365–371 10.1016/S0166-2236(00)01610-6 10906800

[B82] Wenthold J. Petralia S. Blahos J. II Niedzielski S. (1996). Evidence for multiple neurons complexes in hippocampal CA1/CA2. Neurons 76, 1982–1989 10.1523/JNEUROSCI.16-06-01982.1996 PMC6578515 8604042

[B83] Xia J. Wishart D. S. (2011). Web-based inference of biological patterns, functions and pathways from metabolomic data using metaboanalyst. Nat. Protoc. 6, 743–760 10.1038/nprot.2011.319 21637195

[B84] Yamin G. (2009). NMDA receptor-dependent signaling pathways that underlie amyloid beta-protein disruption of LTP in the hippocampus. J. Neurosci. Res. 87, 1729–1736 10.1002/jnr.21998 19170166

